# Association Between Iron Status and Risk of Chronic Kidney Disease in Chinese Adults

**DOI:** 10.3389/fmed.2019.00303

**Published:** 2020-01-08

**Authors:** Yongjian Zhu, Xiaozhuan Liu, Ning Li, Lingling Cui, Xiaofeng Zhang, Xinxin Liu, Kailun Yu, Yao Chen, Zhongxiao Wan, Zengli Yu

**Affiliations:** ^1^School of Public Health, Zhengzhou University, Zhengzhou, China; ^2^College of Food Science and Technology, Henan Agriculture University, Zhengzhou, China

**Keywords:** iron, transferrin, soluble transferrin receptor, chronic kidney disease, eGFR

## Abstract

**Background:** Even though it is well-known that iron deficiency is the result of chronic kidney disease (CKD), whether iron will affect kidney function and disease in the general population is not clear. We thus conducted a nationwide cross-sectional study using data from the China Health and Nutrition Survey (CHNS) to assess the relationship of iron status with estimated glomerular filtration rate (eGFR) and CKD among general adults.

**Methods:** A total of 8,339 adults from the China Health and Nutrition Survey in the wave of 2009 were included to assess the association between iron status and eGFR/CKD. Serum ferritin (SF), transferrin, soluble transferrin receptor (sTfR), and hemoglobin (Hb) were measured. The relationship of iron status and eGFR was evaluated by using multi-variable linear regression model. The effect of iron status on the odds of CKD was calculated by logistic regression model.

**Results:** For the association between iron status and eGFR, every 100 μg/L increase in SF was correlated with 0.26 ml/min per 1.73 m^2^ (95% CI: 0.08–0.44) decrease in eGFR, and every 5 mg/L increase in sTfR was associated with a decrease of 6.00 ml/min per 1.73 m^2^ (95% CI: 3.79–8.21) in eGFR. There were no significant associations between Hb or transferrin with eGFR. For the association between iron status and CKD, every 5 g/L increase in sTfR was associated with an odds ratio of 3.72 (95% CI: 2.16–6.13) for CKD. The concentrations of Hb were associated with the odds of CKD in a U-shaped manner, with the lowest risk in the Hb range of 136–141 g/L. There was a positive correlation between SF concentration and CKD prevalence but not in a dose–response manner. The odds of CKD for participants in the highest tertile increased by 28% (98% CI: 1–63%) compared with those in the lowest tertile.

**Conclusion:** The concentration of SF and sTfR was positively correlated with the odds of CKD, and Hb was associated with the odds of CKD in a U-shaped manner. Further large prospective researches are warranted to confirm these findings.

## Introduction

Affected by the global population aging, chronic kidney disease (CKD), the 17th leading cause of death worldwide (1), is becoming a worldwide public health and can lead to a series of adverse health outcomes including premature death, poor quality of life, and cardiovascular disease (2–4). Moreover, smoking, alcohol use disorder, and obesity are all well-known risk factors for CKD (5–7).

As an oxidative stressor, body iron overload may transform less active free radicals into more active hydroxyl radicals. The activated radicals can have an effect on DNA, proteins, lipids, and metabolite profiles, thus leading to tissue damage and dysfunction (8). On the other hand, excess iron was also associated with inflammation. Both of them contribute to the progression of renal disease (9).

It is well-known that iron depletion is a consequence of CKD, and the supplementation of iron has been widely used as prevention and treatment of anemia in patients with CKD (10). However, it is unclear whether iron will affect kidney function and disease in the general population. Previous studies using experimental models demonstrated that iron can be nephrotoxic through the formation of free radicals (11), even in the absence of systemic overload. Serum ferritin (SF), soluble transferrin receptor (sTfR), hemoglobin (Hb), and transferrin are the most widely used clinical biomarkers of iron. Previous publications have reported the relationships between these markers with hypertension (12), hyperuricemia (13), stroke (14), and diabetes (15). However, there are limited studies evaluating whether iron levels are associated with estimated glomerular filtration rate (eGFR)/CKD in general population. Since CKD is regarded as a risk factor for these aforementioned diseases (4), it is tempting to speculate that the risk factors associated with these disease might possibly be the risk factor for CKD. We thus conducted a nationwide cross-sectional study to assess the relationship between iron status and eGFR/CKD in 8,339 adults in China.

## Methods

### Study Population

The participants were from the China Health and Nutrition Survey (CHNS), which has been elucidated in detail in previous publications (12, 16). Briefly, the samples were selected using a multi-stage random clustering method from nine different provinces (Shandong, Liaoning, Jiangsu, Hunan, Hubei, Henan, Heilongjiang, Guizhou, and Guangxi). According to the level of income (low, medium, and high), the CHNS randomly selected four cities in rural sites and two cities in urban sites from each province with a weighted sampling program. There have been nine waves of surveys since 1989 (i.e., 1989, 1991, 1993, 1997, 2000, 2004, 2006, 2009, and 2011). During each wave, all of the participants underwent a series of examinations. The self-reported questionnaire was used to collected participants' sociodemographic factors and nutritional habits. Blood sample was firstly collected and tested in 2009, and the longitudinal data collected in 2011 and 2015 are being updated. The written informed consent forms were signed by every participant prior to joining our medical screening scheme. The study was approved by the institutional review committees of the University of North Carolina at Chapel Hill, the National Institute of Nutrition and Food Safety, and the Chinese Center for Disease Control and Prevention.

**Figure 1:**
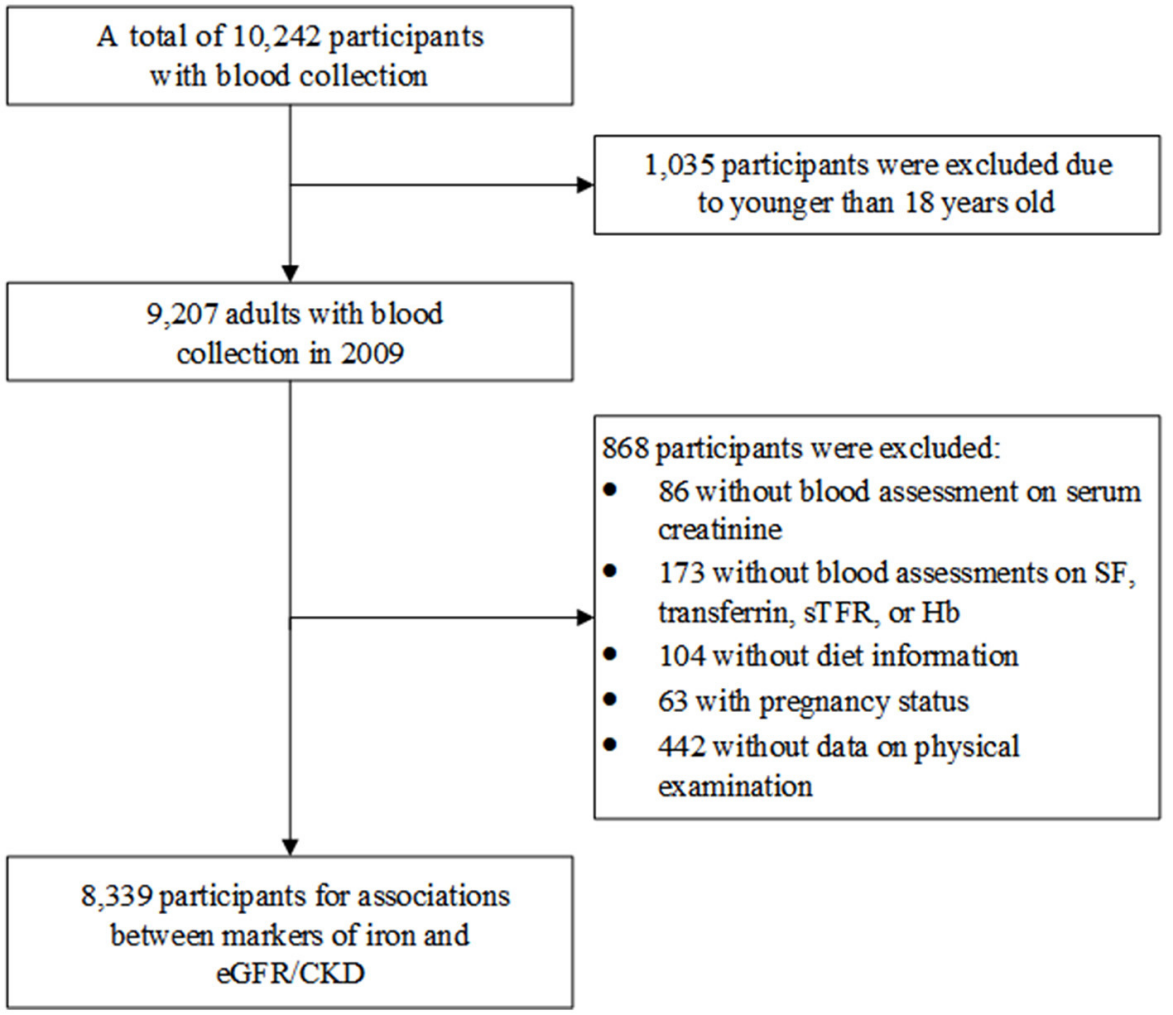
Flowchart of participant selection.

Figure 1 shows the process of participants' selection for this study. We initially selected 9,207 adults (age ≥18 years) who enrolled in 2009 when blood sample was collected for the first time. Subsequently, participants without information of serum creatinine (*n* = 86), iron markers (SF, transferrin, sTfR, or Hb; *n* = 173), diet (*n* = 104); with pregnancy status (*n* = 63); or without physical examination (*n* = 442) were excluded. Consequently, 8,339 participants were finally included to assess the relationship of iron status and eGFR/CKD.

### Laboratory Data Measurement

Blood samples were taken in the morning after at least 12 h of night fasting. As depicted elsewhere (13), all samples were validated and analyzed in accordance with strict quality control standards (17, 18) at the National Center Laboratory (Medical Laboratory Accreditation Certificate ISO 15189:2007) in Beijing. Methods of measuring iron markers have been depicted in detail in previous studies (19, 20). In short, the SF level was measured by radioimmunology at the gamma counter XH-6020 (Northern Institute of Biotechnology, Beijing, China), and serum transferrin and sTfR were detected by nephelometry on a Siemens B-type natriuretic peptide assay (Siemens, Erlangen, Germany). Whole blood Hb concentrations were measured by an LH75 hematology analyzer (Beckman Coulter, Brea, CA, USA). The creatinine concentrations were measured by picric acid method on the Hitachi 7600 (Randox, UK). An eGFR <60 ml/min per 1.73 m^2^ was defined as CKD (21, 22):

$$\begin{array}{l} \text{eGFR}=175\times \text{Sc}{\text{r}}^{-1.234}\times \text{ag}{\text{e}}^{-0.179}\left[\text{if female},\times 0.79\right] \end{array}$$

where Scr is serum creatinine concentration (in mg/dl) and age in years.

### Covariates

Weight was measured to the nearest 0.1 kg by a calibrated beam balance with light clothing. Height was measured to the nearest 0.1 cm using portable SECA meter without shoes. Body mass index (BMI) was calculated as weight (kg) divided by the square of height (m^2^). The structured questionnaires were adopted to collect information on gender, age, educational level, smoking, and drinking habit. Three consecutive 24-h dietary recalls (2 work days and 1 weekend) at the individual level, or weighted food stocks for the same 3 days at the household level were selected to evaluate diet intakes (23). Dietary intake of total energy, carbohydrates, protein, and fat was calculated according to the Chinese Food Composition Table 2004 and 2009 (24, 25).

### Statistical Analyses

All statistical analyses were conducted using R 3.3.2. (R Core Team, Vienna, Austria), and two-sided *P* < 0.05 was considered statistically significant. Descriptive data are expressed as number (percentage) for categorical variables or mean (SD) for continuous variables.

Multivariable linear regression model was adopted to investigate the relationship of iron status and eGFR. We used logistic regression model to calculate the effect of iron status on the odds of CKD. Three models were adopted: Model 1, without any adjustment; Model 2, adjusting for age (continuous, years), gender (female or male), educational level (<6, 6.1–9.0, 9.1–12, or >12 years), smoking habits (never or ever), drinking (yes or no), total energy intake (continuous, kcal), protein intake (continuous, % energy), fat intake (continuous, % energy), and carbohydrate intake (continuous, % energy); Model 3, further adjusting for CKD (not for the relationship between iron status and CKD) and BMI (<25, 18.5–24.9, 25.0–29.9, or ≥30). We used deciles to draw the dose-response relationship between iron status and eGFR/CKD.

Subgroup analyses were also conducted to investigate whether the associations were modified by sex (male or female), age (<60 or ≥60 years), BMI (<25 or ≥25 kg/m^2^), smoking status (never or ever), and drinking (yes or no). Each potential modifier was examined in a separate model by adding a multiplicative interaction term (i.e., potential modifier ^*^ continuous markers of iron).

SF reflects the iron storage pool. sTfR, focusing on both total erythropoiesis and body iron storage, is considered as a better indicator of iron metabolism. In addition, it is not affected by inflammation. It has been suggested that sTfR/ferritin index is a good estimate of body iron (26, 27). We thus conducted a sensitivity analysis using the sTfR/ferritin index to estimate the relationship between iron status and eGFR/CKD.

## Results

Table 1 depicts the general characteristics of included participants. The participants with CKD were older, less likely to have higher education, and less likely to consume alcohol than those without CKD. Moreover, the participants with CKD had higher levels of SF and sTfR, but lower concentration of transferrin and Hb.

**Table 1:** Baseline characteristics of the included study participants.

**Characteristics**	**All participants**	**Non-CKD**	**CKD**	***P***
*N*	8,339	7,711	628	
Age, years	51.0 (15.0)	49.7 (14.4)	66.9 (11.9)	<0.001
**Gender**				0.945
Male	3,915 (46.9%)	3,621 (47.0%)	294 (46.8%)	
Female	4,424 (53.1%)	4,090 (53.0%)	334 (53.2%)	
**Education**				<0.001
<6 years	4,412 (52.9%)	3,983 (51.7%)	429 (68.3%)	
6–9 years	2,360 (28.3%)	2,259 (29.3)	101 (16.1%)	
9–12 years	1,319 (15.8%)	1,236 (16.0%)	83 (13.2%)	
>12 years	248 (3.0%)	233 (3.0%)	15 (2.4)	
**Current smoker**				0.084
Yes	2,593 (31.1%)	2,417 (31.3%)	176 (28.0%)	
No	5,746 (68.9%)	5,294 (68.7%)	452 (72.0%)	
**Alcohol consumption**				<0.001
Yes	2,726 (32.7%)	2,594 (33.6%)	132 (21.0%)	
No	5,613 (67.3%)	5,117 (66.4%)	496 (79.0%)	
BMI, kg/m^2^	23.4 (3.5)	23.4 (3.5)	23.6 (3.8)	0.184
Total energy intake, kcal/day	2,433.5 (781.6)	2440.2 (786.3)	2351.4 (717.5)	<0.001
Carbohydrate intake, % energy	62.7 (12.6)	62.9 (12.6)	60.1 (12.1)	0.006
Protein intake, % energy	12.1 (2.6)	12.1 (2.6)	12.1 (2.7)	0.740
Fat intake, % energy	24.6 (11.8)	24.4 (11.8)	26.8 (11.3)	<0.001
SF, μg/L	137.7 (185. 4)	136.2 (186.1)	156.6 (174.9)	0.008
Transferrin, g/L	2.9 (0.5)	2.9 (0.5)	2.7 (0.6)	<0.001
sTfR, mg/L	1.5 (0.7)	1.5 (0.7)	1.6 (0.6)	<0.001
Hb, g/L	141.4 (20.7)	141.8 (20.4)	136.1 (22.5)	<0.001

*CKD, chronic kidney disease; SF, serum ferritin; sTfR, soluble transferrin receptor; BMI, body mass index; Hb, hemoglobin*.

*The statistical data are shown as means (SDs) for continuous variables and counts (percentages) for categorical variables*.

**Figure 2:**
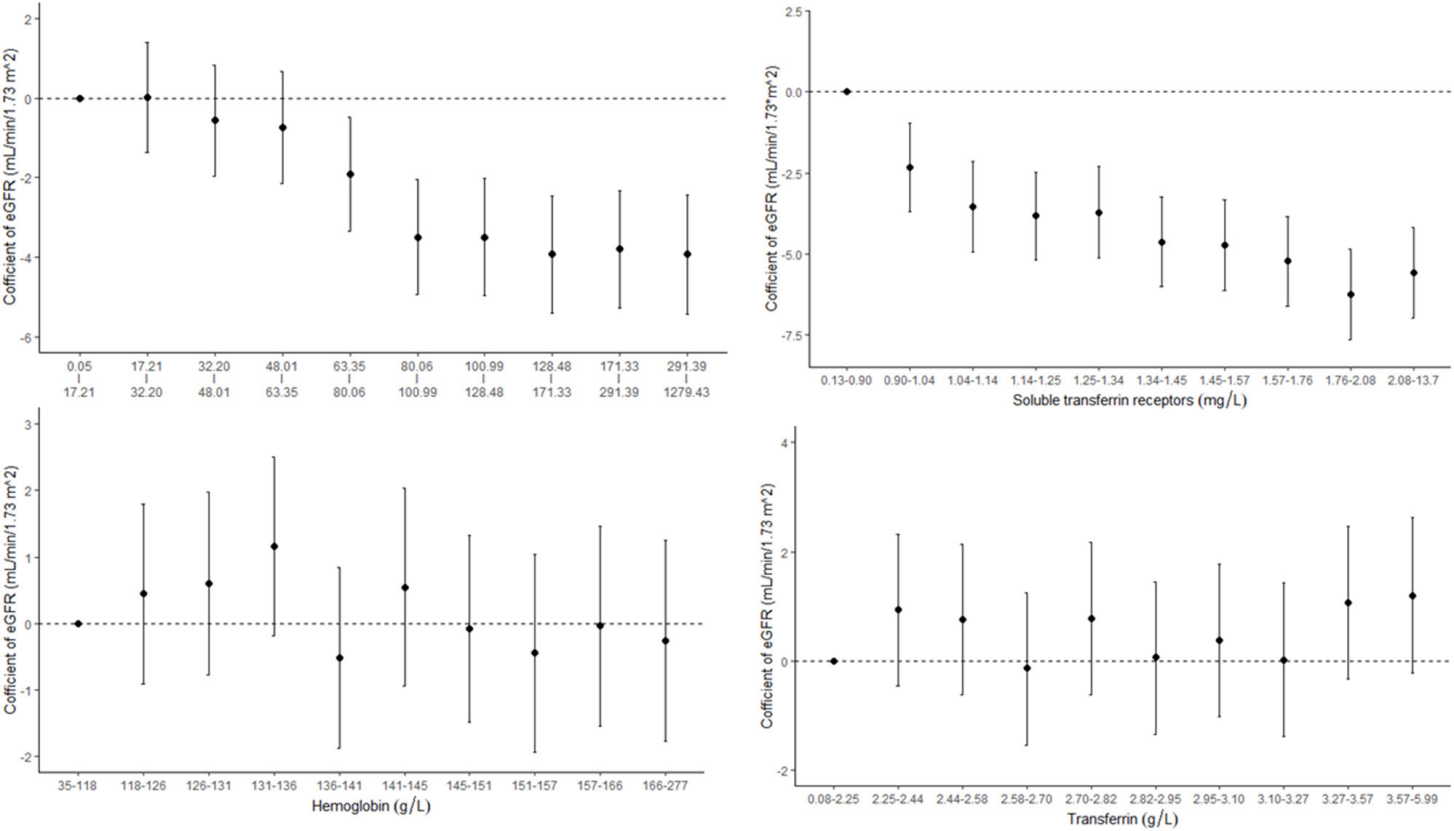
Concentration–response relationships between markers of iron status and estimated glomerular filtration rate (eGFR).

The associations between iron status and eGFR are presented in Table 2 and Figure 2. Both SF and sTfR were negatively associated with eGFR. Results from the adjusted models show that every 100 μg/ml increase in SF was correlated with a decrease of 0.26 ml/min per 1.73 m^2^ (95% CI: 0.08–0.44) in eGFR, and every 5 mg/L increase in sTfR was associated with a decrease of 6.00 ml/min per 1.73 m^2^ (95% CI: 3.79–8.21) in eGFR. There were no significant associations between Hb or transferrin and eGFR.

**Table 2:** Associations of SF, sTfR, Hb, and transferrin levels with eGFR in Chinese adults.

	**Model 1**		**Model 2**		**Model 3**	
	**Coef (95% CI)**	***P***	**Coef (95% CI)**	***P***	**Coef (95% CI)**	***P***
**SERUM FERRITIN**						
Tertile 1st (0.13–52.39 μg/L)	Ref		Ref		Ref	
Tertile 2nd (52.39–115.95 μg/L)	−5.27 (−6.19, −4.34)	<0.001	−2.02 (−2.92, −1.13)	<0.001	−1.98 (−2.79, −1.17)	<0.001
Tertile 3rd (115.95–1279.43 μg/L)	−6.29 (−7.21, −5.37)	<0.001	−3.58 (−4.53, −2.64)	<0.001	−3.24 (−4.11, −2.37)	<0.001
Trend		<0.001		<0.001		<0.001
Every 100 μg/L increase	−0.54 (−0.75, −0.34)	<0.001	−0.33 (−0.52, −0.14)	0.001	−0.26 (−0.44, −0.08)	0.004
**SOLUBLE TRANSFERRIN RECEPTOR**						
1st tertile (0.13–1.18 mg/L)	Ref		Ref		Ref	
2nd tertile (1.18–1.52 mg/L)	−2.64 (−3.57, −1.70)	<0.001	−2.57 (−3.40, −1.73)	<0.001	−2.23 (−2.99, −1.46)	<0.001
3rd tertile (1.52–13.7 mg/L)	−4.77 (−5.69, −3.84)	<0.001	−4.42 (−5.25, −3.59)	<0.001	−3.41 (−4.17, −2.65)	<0.001
Trend		<0.001		<0.001		<0.001
Every 5 mg/L increase	−7.05 (−9.74, −4.36)	<0.001	−8.11 (−10.52, −5.69)	<0.001	−6.00 (−8.21, −3.79)	<0.001
**HEMOGLOBIN**						
1st tertile (35.00–132.00 g/L)	Ref		Ref		Ref	
2nd tertile (132.00–149.00 g/L)	1.31 (0.37, 2.25)	0.007	0.81 (−0.02, 1.70)	0.055	0.05 (−0.74, 0.84)	0.910
3rd tertile (149.00–277.00 g/L)	2.58 (1.63, 3.52)	<0.001	0.08 (−0.94, 1.10)	0.881	−0.64 (−1.59, 0.30)	0.181
Trend		<0.001		0.880		0.181
Every 10 g/L increase	0.59 (0.41, 0.77)	<0.001	0.12 (−0.07, 0.31)	0.220	−0.01 (−0.18, 0.17)	0.933
**TRANSFERRIN**						
1st tertile (0.08–2.62 g/L)	Ref		Ref		Ref	
2nd tertile (2.61–3.04 g/L)	2.15 (1.21, 3.09)	<0.001	0.09 (−0.76, 0.94)	0.830	−0.19 (−0.96, 0.58)	0.627
3rd tertile (3.04–5.99 g/L)	3.57 (2.65, 4.50)	<0.001	0.48 (−0.37, 1.32)	0.267	0.17 (−0.60, 0.95)	0.659
Trend		<0.001		0.254		0.607
Every 5 g/L increase	15.62 (12.17, 19.06)	<0.001	3.24 (0.09, 6.40)	0.044	1.94 (−0.97, 4.84)	0.191

*Model 1 without any adjustments; model 2 adjusted for age, gender, nationality (Han or others), education (6, 6.1–9.0, 9.1–12, or >9 years), smoking status (current or not current), alcohol consumption (yes or no), total energy intake (quartile), protein intake (quartile), fat intake (quartile), and carbohydrate intake (quartile); model 3 adjusted as for model 2 plus BMI (<18.5, 18.5–24.9, 25.0–29.9, or ≥30 kg/m^2^) and CKD*.

**Figure 3:**
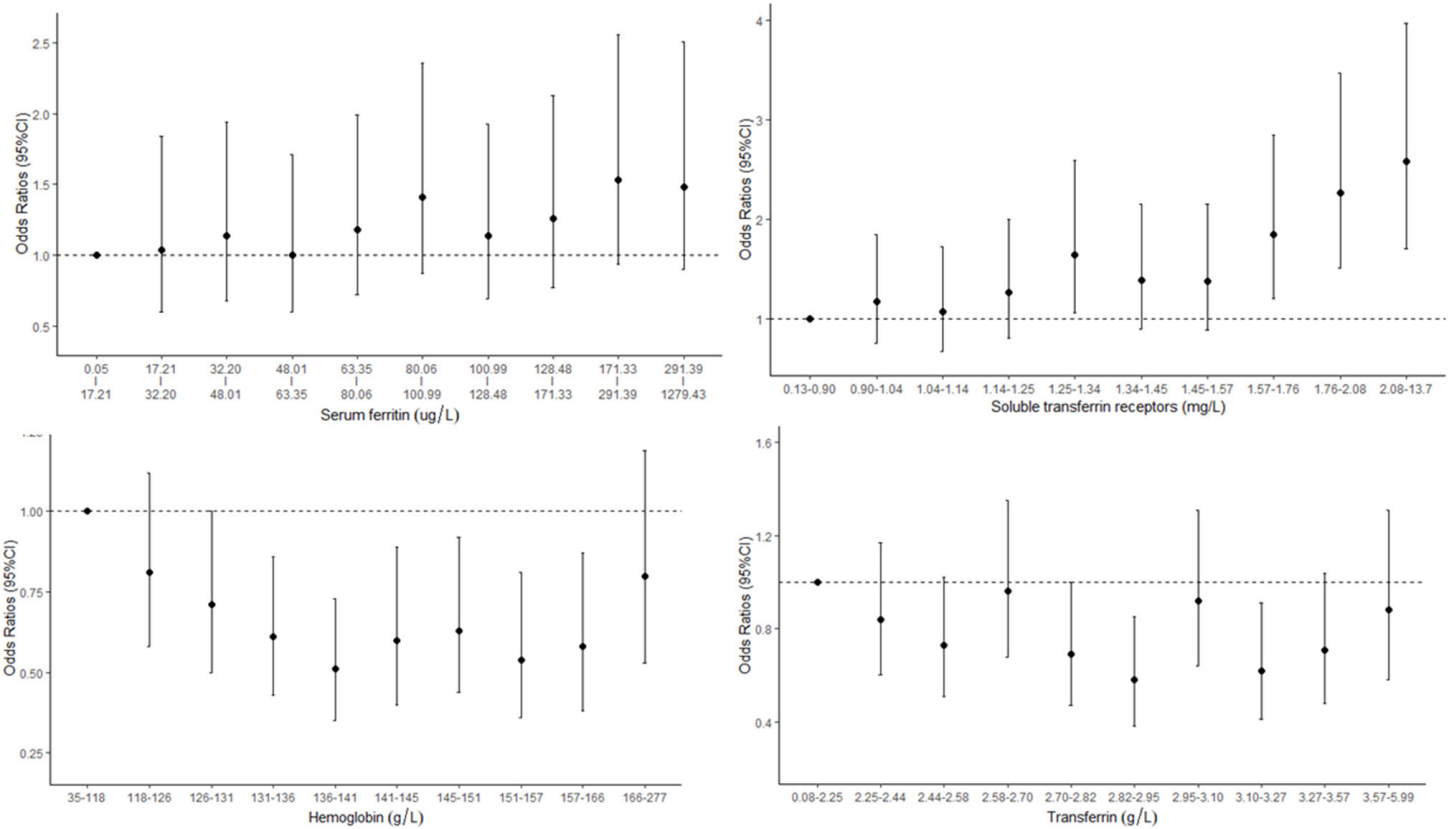
Concentration–response relationships between markers of iron status and odds of chronic kidney disease (CKD).

The associations between iron status and odds of CKD are presented in Table 3 and Figure 3. The concentrations of sTfR were positively correlated with the odds of CKD with a dose–response trend. Every 5 mg/L increase in sTfR was associated with an odds ratio of 3.72 with 95% CI of 2.16–6.13. The concentrations of Hb were associated with the odds of CKD in a U-shaped manner, with the lowest risk in the Hb range of 136–141 g/L. The levels of SF were positively correlated with the odds of CKD but not in a dose–response manner. The odds of CKD for participants in the highest tertile increased by 28% (98% CI: 1–63%) compared with those in the lowest tertile.

**Table 3:** Associations of SF, sTfR, Hb, and transferrin levels with the odds of CKD in Chinese adults.

	**Model 1**		**Model 2**		**Model 3**	
	**HR (95% CI)**	***P***	**HR (95% CI)**	***P***	**HR (95% CI)**	***P***
**SERUM FERRITIN**						
Tertile 1st (0.13–52.39 μg/L)	Ref		Ref		Ref	
Tertile 2nd (52.39–115.95 μg/L)	1.93 (1.55, 2.42)	<0.001	1.15 (0.90, 1.46)	0.263	1.12 (0.88, 1.43)	0.346
Tertile 3rd (115.95–1279.43 μg/L)	2.09 (1.69, 2.61)	<0.001	1.31 (1.03, 1.67)	0.027	1.28 (1.01, 1.63)	0.047
Trend		<0.001		0.034		0.050
Every 100 μg/ml increase	1.05 (1.01, 1.09)	0.008	1.05 (1.00, 1.10)	0.062	1.04 (0.99, 1.09)	0.122
**SOLUBLE TRANSFERRIN RECEPTOR**						
1st tertile (0.13–1.18 mg/L)	Ref		Ref		Ref	
2nd tertile (1.18–1.52 mg/L)	1.29 (1.03, 1.61)	0.025	1.34 (1.05, 1.70)	0.015	1.33 (1.05, 1.69)	0.017
3rd tertile (1.52–13.7 mg/L)	1.90 (1.55, 2.33)	<0.001	1.95 (1.57, 2.45)	<0.001	1.93 (1.55, 2.42)	<0.001
Trend		<0.001		<0.001		<0.001
Every 5 mg/L increase	2.25 (1.42, 3.47)	<0.001	3.66 (2.13, 6.01)	<0.001	3.72 (2.16, 6.13)	<0.001
**HEMOGLOBIN**						
1st tertile (35.00–132.00 g/L)	Ref		Ref		Ref	
2nd tertile (132.00–149.00 g/L)	0.65 (0.54, 0.79)	<0.001	0.69 (0.56, 0.85)	<0.001	0.68 (0.55, 0.84)	<0.001
3rd tertile (149.00–277.00 g/L)	0.52 (0.42, 0.63)	<0.001	0.73 (0.56, 0.94)	0.014	0.69 (0.53, 0.89)	0.005
Trend		<0.001		0.008		0.002
Every 10 g/L increase	0.87 (0.84, 0.91)	<0.001	0.94 (0.89, 0.98)	0.008	0.93 (0.88, 0.97)	0.002
**TRANSFERRIN**						
1st tertile (0.08–2.62 g/L)	Ref		Ref		Ref	
2nd tertile (2.61–3.04 g/L)	0.64 (0.53, 0.78)	<0.001	0.89 (0.72, 1.10)	0.296	0.88 (0.71, 1.09)	0.240
3rd tertile (3.04–5.99 g/L)	0.51 (0.42, 0.63)	<0.001	0.85 (0.68, 1.06)	0.144	0.82 (0.66, 1.02)	0.082
Trend		<0.001		0.140		0.079
Every 5 g/L increase	0.05 (0.02, 0.11)	<0.001	0.45 (0.19, 1.06)	0.068	0.39 (0.16, 0.93)	0.035

*Model 1 without any adjustments; model 2 adjusted for age, gender, education (<6, 6.1–9.0, 9.1–12, or >12 years), smoking status (never or ever), alcohol consumption (yes or no), total energy intake (quartile), protein intake (quartile), fat intake (quartile), and carbohydrate intake (quartile); model 3 adjusted as for model 2 plus BMI (<18.5, 18.5–24.9, 25.0–29.9, or ≥30 kg/m^2^)*.

Subgroup and sensitivity analyses generally yielded similar results (Tables S1–S5).

## Discussion

To the best of our knowledge, this is the first study to investigate the relationship between SF, transferrin, sTRF, and Hb levels with eGFR and odds of CKD in the same population in general Chinese population. We found that both SF and sTfR were negatively correlated with eGFR. Correspondingly, SF and sTfR were positively associated with the prevalence of CKD. Moreover, we also observed a U-shaped association between Hb and the prevalence of CKD.

Even though it is well-known that iron deficiency is the result of CKD, whether iron status is associated with the risk of CKD in general population is unclear. Several studies previously investigated the associations between iron status and CKD/renal function in general population with insosistent results (27–31), and most of them generally focused on the markers of iron with a single type. We found that participants in the highest tertile of SF had increased risk of CKD than those in the lowest tertile, which was comparable with previous studies (27, 29–31). However, such an association disappeared when the SF was considered as the continuous variable. The exact causes for this phenomenon are not clear, but we speculate that the use of category variable may result in information loss and increased uncertainty. Moreover, the concentration-response association with SF decile also suggested that the relationships were non-significant. It is difficult to explain this phenomenon, but the non-linearity relationship might also be ascribed to the population heterogeneities and other unidentified confounders. Moreover, the stratified analysis found a positive association between SF and CKD among participants younger than 60 years old but not among those older than 60 years old. The possible reason for the modification effect might be partly due to decreased responsiveness to autonomic nervous system stimuli among elder participants.

sTRF, uninfluenced by inflammation degree, has been identified as a better indicator of iron deficiency because it helps to diagnose iron deficiency that coexists with anemia of chronic disorders. Unfortunately, a limited number of studies investigated the association between transferrin/sTRF/Hb and CKD. However, our finding of the positive association between sTfR and CKD is consistent with Alam et al., who observed high sTRF levels in patients with CKD than those without CKD (27). This association can also be comparable with previous observations that increased level of sTfR had adverse effects on different health outcomes, including diabetes or insulin resistance (15, 32), iron-deficiency anemia (33), and obesity (34). In this study, we also found a U-shaped relationship of Hb and CKD, which is comparable with findings from other health outcomes, such as mortality (35), birth outcome (36), and stroke severity (14).

The potential mechanism for the relationship of iron status and CKD/eGFR is unclear. It appears that inflammation and oxidative stress are two of the key factors linking the association of iron with CKD. SF, an acute phase protein, was associated with systemic inflammation and total body iron storage (37). Previous studies have discovered an association between iron status and inflammation in early stages of CKD. Low levels of Hb may lead to reduced oxygen carrying capacity and subsequently cause kidney injury. On the other hand, Hb is highly correlated with increased blood viscosity in patients with high hematocrit. High blood viscosity increases blood flow resistance. This may lead to reduced oxygen delivery, which increases the risk of regional ischemic infarction and thereby causes increased risk of CKD. The underlying mechanism of the association between sTfR and CKD might also be ascribed to obesity, which is a main risk factor for CKD (7). Obesity-induced inflammation can increase the synthesis of hepcidin (38), which is a vital hormone in iron homoeostasis. It also prevents the release of iron from enterocytes and the reticuloendothelial system, leading to functional iron deficiency (39). In this context, the cellular transferrin receptor expression may increase. Thus, high sTfR concentration may reflect chronic pathologies such as obesity, and they may therefore be associated with increased risk of CKD development.

Our study has several important strengths. First is the population-based, large-scale, national study design. The CHNS selected a large group of provinces, covering about 56% of the Chinese population, and these selected provinces represented a wide range of demographic and economic disparities in China (40). Another strength is adjusting the detailed information on socio-demographic and lifestyle variables.

The study also has some limitations. First, the cross-sectional study design makes it impossible for us to investigate the causal association between iron status and eGFR/CKD. It is also possible that the association is due to impaired iron absorption and/or having received more iron given anemia among patients with CKD. Further prospective cohort studies are warranted to verify the association. Second, we lack information on economic status. However, we adjusted the effect of education, which is an important indicator of individuals' income. Third, we excluded 868 (9.43%) adults with missing information in our data analysis. Potential selection bias cannot be ruled out. However, there is no evidence suggesting that the participants who were included and excluded had significantly different levels of SF (137.72 vs. 141.04 μg/L), transferrin (2.87 vs. 2.94 g/L), sTfR (1.47 vs. 1.47 mg/L), or Hb (141.38 vs. 140.98 g/L).

In conclusion, we found that SF and sTfR were positively correlated with the prevalence of CKD, and Hb was negatively correlated with the prevalence of CKD in the general population. Further prospective studies are warranted to verify this causal association in populations with high risk of CKD, as well as to clarify the potential mechanisms.

## Data Availability Statement

The datasets generated for this study are available on request to the corresponding author.

## Ethics Statement

The studies involving human participants were reviewed and approved by the University of North Carolina at Chapel Hill, and the National Institute of Nutrition and Food Safety and the Chinese Centre for Disease Control and Prevention. The patients/participants provided their written informed consent to participate in this study.

## Author Contributions

YZ and ZY were the project lead for the current study. XiaL, NL, and LC searched the database and extracted data. YZ, XZ, and XinL conducted data and statistical analysis. YZ wrote the manuscript. KY and YC provided significant advice about the manuscript. ZY and ZW reviewed and revised the manuscript. All authors read and approved the final manuscript.

### Conflict of Interest

The authors declare that the research was conducted in the absence of any commercial or financial relationships that could be construed as a potential conflict of interest.
