# Supplementary material for: Association Between Iron Status and Risk of Chronic Kidney Disease in Chinese Adults
Source: Front Med (Lausanne). 2020 Jan 8;6:303. doi: 10.3389/fmed.2019.00303 (PMC6961557; doi:10.3389/fmed.2019.00303)
Supplement: Supplementary file 1 [file Data_Sheet_1.pdf]

Table S1. The stratified analyses of associations between Serum ferritin and eGFR/CKD by Covariates at baseline

	Case/total	eGFR		CKD	
		**Coef (95% CI)**	***P***	**HR (95% CI)**	***P***
**Sex**			0.243*		0.637*
Male	294/3,915	-0.12 (-0.36, 0.12)	0.326	1.03 (0.96, 1.09)	0.422
Female	334/4,424	-0.46 (-0.78, -0.14)	0.005	1.06 (0.97, 1.14)	0.173
**Age**			0.214*		0.037*
<60	177/6,016	-0.24 (-0.47, -0.01)	0.042	1.11 (1.03, 1.18)	0.004
≥ 60	451/2,323	-0.15 (-0.41, 0.10)	0.232	0.99 (0.92, 1.06)	0.759
**BMI (kg/m^2)**			0.547*		0.935*
<25	414/5,848	-0.08 (-0.31, 0.15)	0.492	1.04 (0.96, 1.11)	0.328
≥ 25	214/2,491	-0.37 (-0.62, -0.11)	0.005	1.05 (0.98, 1.12)	0.184
**Smoking status**			0.756*		0.909*
Ever	176/2,593	-0.17 (-0.49, 0.15)	0.303	1.04 (0.96, 1.12)	0.352
Never	452/5,746	-0.27 (-0.49, -0.05)	0.015	1.04 (0.98, 1.11)	0.167
**Drinking**			0.228*		0.591*
Yes	132/2,726	-0.20 (-0.51, 0.10)	0.194	1.03 (0.94, 1.12)	0.458
No	496/5,613	-0.29 (-0.51, -0.06)	0.012	1.05 (0.99, 1.11)	0.112

*The P value for interaction terms between iron status and sex, age, BMI, smoking, and drinking

Table S2. The stratified analyses of associations between soluble transferrin receptor and eGFR/CKD by Covariates at baseline

	Case/total	eGFR		CKD	
		**Coef (95% CI)**	***P***	**HR (95% CI)**	***P***
**Sex**			0.073*		0.275*
Male	294/3,915	-11.23 (-16.18, -6.28)	<0.001	6.03 (2.18, 16.18)	<0.001
Female	334/4,424	-4.34 (-6.44, -2.25)	<0.001	3.12 (1.55, 5.71)	<0.001
**Age**			<0.001*		0.577*
<60	177/6,016	-4.86 (-7.50, -2.22)	<0.001	3.55 (1.70, 6.64)	<0.001
≥ 60	451/2,323	-11.64 (-15.49, -7.78)	<0.001	4.31 (1.76, 10.43)	0.001
**BMI (kg/m^2)**			0.052*		0.186*
<25	414/5,848	-5.78 (-8.48, -3.09)	<0.001	2.73 (1.38, 4.99)	0.002
≥ 25	214/2,491	-6.77 (-10.56, -2.99)	<0.001	6.99 (2.51, 18.11)	<0.001
**Smoking status**			0.002*		0.029*
Ever	176/2,593	-11.86 (-18.37, -5.35)	<0.001	11.99 (3.60, 41.76)	<0.001
Never	452/5,746	-5.00 (-7.12, -2.88)	<0.001	2.78 (1.42, 4.99)	0.001
**Drinking**			0.147*		0.042*
Yes	132/2,726	-10.27 (-16.73, -3.80)	0.002	14.84 (3.28, 58.82)	<0.001
No	496/5,613	-5.13 (-7.24, -3.02)	<0.001	3.06 (1.65, 5.31)	<0.001

*The P value for interaction terms between iron status and sex, age, BMI, smoking, and drinking

Table S3. The stratified analyses of associations between hemoglobin and eGFR/CKD by Covariates at baseline

	Case/total	eGFR		CKD	
		**Coef (95% CI)**	***P***	**HR (95% CI)**	***P***
**Sex**			0.150*		0.119*
Male	294/3,915	0.01 (-0.30, 0.31)	0.973	0.96 (0.89, 1.02)	0.186
Female	334/4,424	0.05 (-0.15, 0.24)	0.640	0.89 (0.83, 0.96)	0.002
**Age**			<0.001*		0.586*
<60	177/6,016	-0.19 (-0.41, 0.04)	0.106	0.94 (0.86, 1.03)	0.179
≥ 60	451/2,323	0.46 (0.23, 0.69)	<0.001	0.92 (0.86, 0.97)	0.004
**BMI (kg/m^2)**			0.590*		0.589*
<25	414/5,848	0.03 (-0.19, 0.25)	0.781	0.95 (0.89, 1.01)	0.075
≥ 25	214/2,491	-0.03 (-0.31, 0.25)	0.824	0.88 (0.81, 0.96)	0.005
**Smoking status**			0.453*		0.744*
Ever	176/2,593	0.06 (-0.35, 0.46)	0.777	0.93 (0.85, 1.02)	0.144
Never	452/5,746	0.01 (-0.17, 0.19)	0.946	0.92 (0.87, 0.98)	0.008
**Drinking**			0.128*		0.722*
Yes	132/2,726	-0.06 (-0.45, 0.32)	0.739	0.96 (0.87, 1.07)	0.483
No	496/5,613	0.02 (-0.16, 0.20)	0.821	0.92 (0.87, 0.97)	0.003

*The P value for interaction terms between iron status and sex, age, BMI, smoking, and drinking

Table S4. The stratified analyses of associations between transferrin and eGFR/CKD by Covariates at baseline

	Case/total	eGFR		CKD	
		**Coef (95% CI)**	***P***	**HR (95% CI)**	***P***
**Sex**			0.815*		0.712*
Male	294/3,915	5.66 (0.38, 10.94)	0.036	0.44 (0.12, 1.60)	0.214
Female	334/4,424	-1.81 (-4.89, 1.27)	0.249	0.38 (0.12, 1.21)	0.102
**Age**			<0.001*		0.757*
<60	177/6,016	1.79 (-1.84, 5.41)	0.334	0.61 (0.14, 2.53)	0.497
≥ 60	451/2,323	2.22 (-2.04, 6.48)	0.307	0.34 (0.11, 1.01)	0.053
**BMI (kg/m^2)**			0.593*		0.799*
<25	414/5,848	0.50 (-3.20, 4.19)	0.793	0.33 (0.11, 0.96)	0.044
≥ 25	214/2,491	6.44 (1.96, 10.92)	0.005	0.58 (0.13, 2.60)	0.475
**Smoking status**			0.073*		0.099*
Ever	176/2,593	5.03 (-1.85, 11.90)	0.152	1.49 (0.27, 7.88)	0.639
Never	452/5,746	0.42 (-2.55, 3.39)	0.781	0.24 (0.09, 0.67)	0.007
**Drinking**			0.010*		0.045*
Yes	132/2,726	4.25 (-2.25, 10.74)	0.200	1.81 (0.29, 10.71)	0.519
No	496/5,613	0.44 (-2.57, 3.44)	0.777	0.26 (0.10, 0.71)	0.008

*The P value for interaction terms between iron status and sex, age, BMI, smoking, and drinking

Table S5. Associations of sTFR/ferritin ratio with eGFR/CKD in Chinese adults

	eGFR		CKD	
	Coef (95% CI)	*P*	HR (95% CI)	*P*
sTFR/ferritin ratio				
Tertile 1st	Ref		Ref	
Tertile 2nd	-0.96(-1.73, -0.18)	0.015	1.14 (0.92, 1.43)	0.232
Tertile 3rd	-1.46 (-2.27, -0.65)	<0.001	1.58 (1.26, 1.98)	<0.001
Trend		0.002		<0.001
Every 10 increase	-0.11(-2.95, 2.73)	0.940	1.65 (0.55, 3.24)	0.238

[a]adjusted for age, gender, nationality (Han or others), education (6 years, 6.1–9.0 years, 9.1-12 years, or >9 years), smoking status (current or not current), alcohol consumption (yes or no), total energy intake (quartile), protein intake (quartile), fat intake (quartile), carbohydrate intake (quartile) BMI (<18.5 kg/m2, 18.5–24.9 kg/m^2, 25.0–29.9 kg/m^2, or ≥30 kg/m^2), and CKD (not for associations between sTFR/ferritin and CKD)
